# Spatial transcriptomics implicates impaired BMP signaling in NF1 fracture pseudarthrosis in murine and patient tissues

**DOI:** 10.1172/jci.insight.176802

**Published:** 2024-07-11

**Authors:** Jonathan J. Rios, Conan Juan, John M. Shelton, Nandina Paria, Ila Oxendine, Meghan Wassell, Yared H. Kidane, Reuel Cornelia, Elise C. Jeffery, David A. Podeszwa, Simon J. Conway, Carol A. Wise, Robert J. Tower

**Affiliations:** 1 Center for Translational Research, Scottish Rite for Children, Dallas, Texas, USA.; 2 McDermott Center for Human Growth and Development,; 3 Department of Pediatrics,; 4 Department of Orthopaedic Surgery,; 5 Simmons Comprehensive Cancer Center,; 6 Department of Surgery,; 7 Department of Internal Medicine,; 8 Department of Pathology, and; 9 Children’s Research Institute, University of Texas Southwestern Medical Center, Dallas, Texas, USA.; 10 Department of Orthopaedics, Scottish Rite for Children, Dallas, Texas, USA.; 11 Wells Center for Pediatric Research, Indiana University School of Medicine, Indianapolis, Indiana, USA.

**Keywords:** Bone biology, Bioinformatics, Bone disease, Genetic diseases

## Abstract

The neurofibromatosis type 1 (NF1) RASopathy is associated with persistent fibrotic nonunions (pseudarthrosis) in human and mouse skeletal tissue. Here, we performed spatial transcriptomics to define the molecular signatures occurring during normal endochondral healing following fracture in mice. Within the control fracture callus, we observed spatially restricted activation of morphogenetic pathways, such as TGF-β, WNT, and BMP. To investigate the molecular mechanisms contributing to *Nf1*-deficient delayed fracture healing, we performed spatial transcriptomic analysis on a *Postn*-cre;*Nf1*^fl/–^ (*Nf1*^Postn^) fracture callus. Transcriptional analyses, subsequently confirmed through phospho-SMAD1/5/8 immunohistochemistry, demonstrated a lack of BMP pathway induction in *Nf1^Postn^* mice. To gain further insight into the human condition, we performed spatial transcriptomic analysis of fracture pseudarthrosis tissue from a patient with NF1. Analyses detected increased MAPK signaling at the fibrocartilaginous-osseus junction. Similar to that in the *Nf1*^Postn^ fracture, BMP pathway activation was absent within the pseudarthrosis tissue. Our results demonstrate the feasibility of delineating the molecular and tissue-specific heterogeneity inherent in complex regenerative processes, such as fracture healing, and reconstructing phase transitions representing endochondral bone formation in vivo. Furthermore, our results provide in situ molecular evidence of impaired BMP signaling underlying NF1 pseudarthrosis, potentially informing the clinical relevance of off-label BMP2 as a therapeutic intervention.

## Abstract 2

We demonstrate how spatial transcriptomics applied to mouse and human disease specimens can delineate the molecular pathogenesis of bone fracture healing defects associated with Neurofibromatosis.

## Introduction

Neurofibromatosis type 1 (NF1; MIM#162200) is an uncommon RASopathy caused by mutations in the *NF1* tumor-suppressor gene, which predisposes patients to pleiotropic secondary sequelae, including skeletal manifestations (1). Children with NF1 may develop long bone dysplasia that is at risk of fracture and persistent fibrous nonunion (pseudarthrosis). Treatment of pseudarthroses includes resection, stabilization, and the addition of bone graft as well as treatment with recombinant human bone morphogenetic protein 2 (rhBMP2) (2). However, clinical benefit of the off-label use of rhBMP2 is uncertain, and both refracture and subsequent amputation persist in this patient population (3, 4).

Fracture pseudarthroses in children with NF1 were previously associated with somatic *NF1* gene mutations (5–7). Neurofibromin, the protein encoded by the *NF1* gene, is a RAS GTPase, and somatic *NF1* mutation (i.e., loss of heterozygosity) leads to aberrant activation of the RAS/MEK/ERK (MAPK) signaling pathway that has been described histologically in patient bone lesions (8, 9). More recently, molecular analyses of cultured patient-derived primary cells support activation of the MAPK signaling pathway in the pathogenesis of fracture pseudarthroses (6, 7). However, the effect of such dysregulation on the endochondral fracture healing process in vivo remains unclear.

Genetically engineered mouse models (GEMMs) have provided important insights into the role of *Nf1* in osteolineage cell differentiation and skeletal development, including in both the bone marrow and periosteal niches (7, 10–14). These studies reproducibly demonstrate how loss of *Nf1* inhibits osteogenic differentiation, leading to impaired skeletal development. Surprisingly, in vitro osteogenic differentiation was not rescued by treating *Nf1*-deficient osteoprogenitor cells with BMP or the WNT agonist osteolectin (7, 12). Similar studies evaluating how loss of *Nf1* affects fracture healing have been limited due to, at least in part, the lack of understanding regarding the skeletal niche responsible for pseudarthroses in patients. Using a cre-expressing adenovirus to broadly delete *Nf1* throughout all cell populations at the fracture site, healing was markedly delayed compared with that of control mice (15). As the periosteum has been repeatedly implicated in the pathogenesis of NF1 pseudarthrosis, skeletal development and fracture healing were previously evaluated following conditional deletion of *Nf1* in the bone periosteum of *Postn*-cre;*Nf1*^fl/–^ (herein called *Nf1*^Postn^) mice. Compared with control mice, skeletal development was impaired in *Nf1*^Postn^ mice (14). Similarly, fracture healing was delayed compared with fractures in control mice, with only 20% of *Nf1*^Postn^ fractures healing after 4 weeks compared with 80% in control mice. Thus, *Nf1*^Postn^ mice faithfully recapitulate the periosteal nature of delayed fracture healing that is observed in humans. Despite robust evidence for a critical role of *Nf1* in regulating osteoblast differentiation and skeletal development, no study has thus far evaluated the molecular basis of delayed fracture healing in any *Nf1* GEMMs.

Spatial transcriptomic analyses of tissue specimens enable the unbiased in situ profiling of molecular heterogeneity within complex tissues as well as contextualization of dysregulation associated with disease states (16–18). Application of spatial transcriptomics has been, for the most part, restricted to soft tissue pathologies, while the suitability of such an approach to mineralized tissues has been limited. Advancing this technique to mature skeletal specimens requires decalcification prior to sectioning of FFPE tissues. If successful, however, spatial transcriptomics has the potential to reveal the dynamic molecular regulation of endochondral bone formation and regeneration in situ. Moreover, analyses of preclinical GEMMs and integration with analyses of patient specimens may further define the tissue-specific context of complex skeletal pathologies.

In the present study, we utilized spatial transcriptomics to spatially define the molecular signatures across endochondral bone formation following fracture in mice. These analyses demonstrate impaired BMP and WNT signaling in *Nf1*^Postn^ fractures, while TGF-β signaling remained intact. Spatial transcriptomic analysis of a specimen from a patient with NF1 confirmed tissue-specific impairment in BMP signaling in the pathogenesis of fracture pseudarthrosis. Our results provide a dynamic cellular context to the molecular dysregulation associated with somatic fracture healing defects in children with NF1 and further demonstrate the feasibility of spatial transcriptomic analyses on mouse and human fracture bone to elucidate disease processes in preclinical and clinical mineralized tissues.

## Results

### Localization and characterization of fracture-associated spatial clusters.

**Figure 1:**
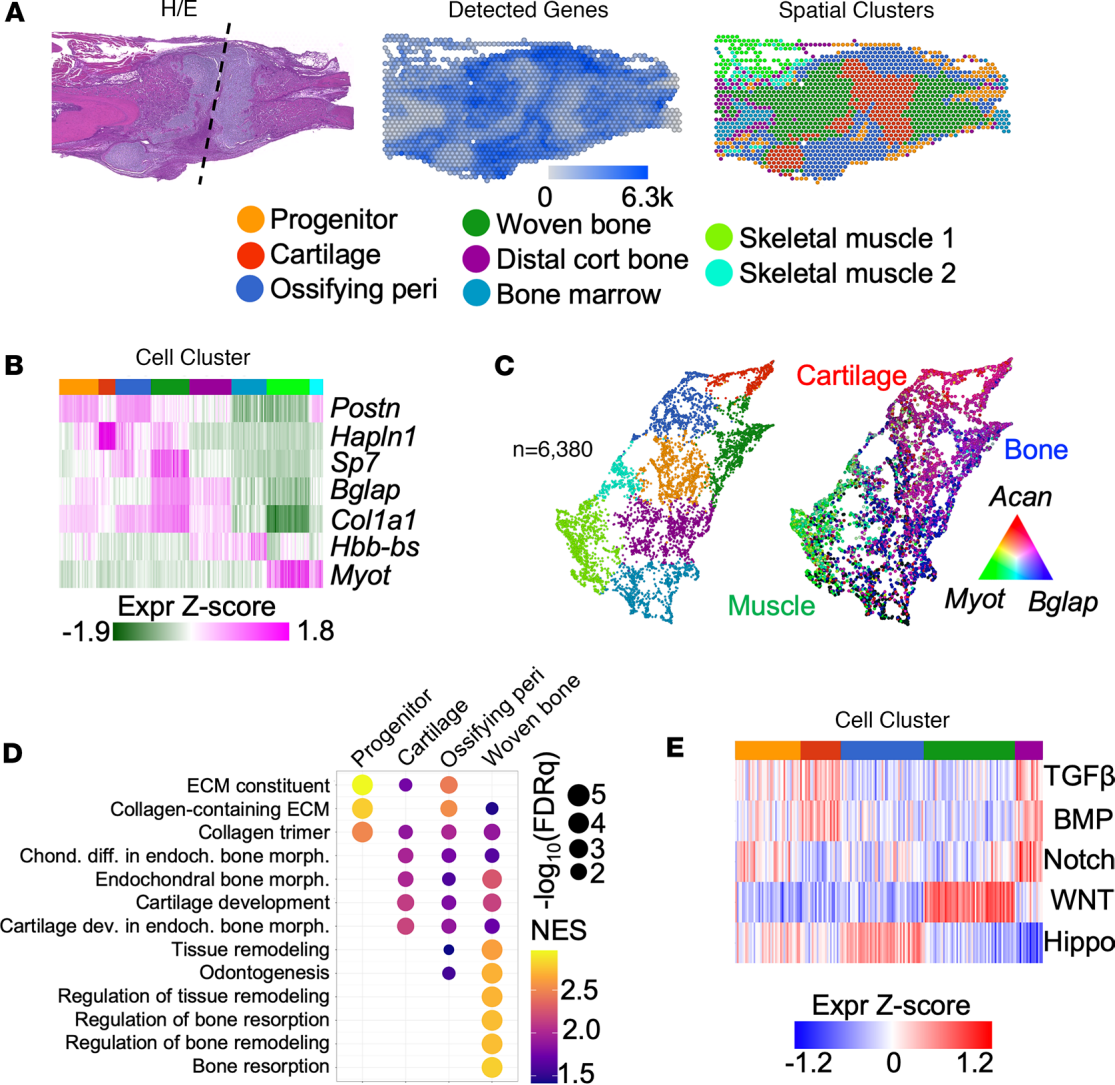
Spatial transcriptomic analysis of fracture healing in mice. (**A**) Spatial histology stained by H&E (left) and localization of the number of detected genes (middle) or computationally defined cell clusters (right) within the control fracture callus. Dashed line indicates fracture plane. (**B**) Heatmap of selected biomarker gene expression across each graph-based cluster. (**C**) UMAP of spatial clusters and identification of muscle (green, *Myot*), cartilage (red, *Acan*), and osteogenic/skeletal (blue, *Bglap*) clusters. (**D**) GSEA demonstrating pathways significantly enriched in the regenerative spatial clusters. (**E**) Heatmap depicting expression of genes associated with indicated signaling pathways across skeletal spatial clusters.

While previous transcriptome studies have sought to unravel the biological complexity of fracture healing (19–21), the fracture callus is a heterogenous tissue with a 3D architecture that is critical for its mechanical function and biological regulation. To overcome this lack of spatial information, we performed spatial transcriptomic analyses of distal tibia fractures from adult control (Figure 1A) and *Nf1*^Postn^ mice collected 10 days after injury (Supplemental Table 1; supplemental material available online with this article; https://doi.org/10.1172/jci.insight.176802DS1). Loss of *Nf1* in *Nf1*^Postn^ mice was previously associated with impaired skeletal progenitor differentiation and delayed fracture healing (14). To minimize the confounding effects of these biologic variables on the identification and characterization of tissue types within the fracture calluses (22), spatial expression data from both the control and *Nf1*^Postn^ callus were integrated and harmonized for initial cluster detection and characterization. The transcriptome of each spot was used for graph-based clustering analysis following dimensional reduction by principal component analysis. This method detected 8 transcriptionally unique spatial clusters within the fracture calluses. To classify the identities of these clusters, we investigated their histologic distribution within the control fracture (Figure 1A) as well as the expression of biomarkers associated with each cluster in the harmonized data set (Figure 1B and Supplemental Table 2). Using the uniform manifold approximation and projection (UMAP) analysis to discern the relative relatedness among clusters, we identified distinct cartilage, bone, and muscle cell types marked by *Acan*, *Bglap*, and *Myot* expression, respectively (Figure 1C and Supplemental Table 3).

Having identified multiple clusters and their associated biomarkers, we next evaluated more broadly the expression of molecular pathways within the skeletal clusters implicated in fracture healing (progenitor, cartilage, ossifying perichondrium, and newly formed woven bone) using gene set enrichment analysis (GSEA) (Supplemental Tables 4–7). GSEA analysis showed that increased expression of collagen and extracellular matrix (ECM) gene sets was most strongly associated with the progenitor cluster, followed by cartilage and endochondral morphogenesis gene sets in the cartilage and ossifying perichondrium clusters, and bone mineralization and remodeling gene sets in the newly formed woven bone (Figure 1D). Finally, we investigated the relative expression of genes associated with activation of morphogenetic pathways implicated in skeletal development and fracture healing. Expression of these pathways was generally restricted to one or few clusters, demonstrating the tissue-specific expression of these pathways during fracture healing (Figure 1E). These results demonstrate the ability of spatial transcriptomics to spatially distinguish unique transcriptional signatures associated with diverse cell populations in a tibia fracture mouse model.

### Spatially restricted signaling gradients within the fracture callus.

**Figure 2:**
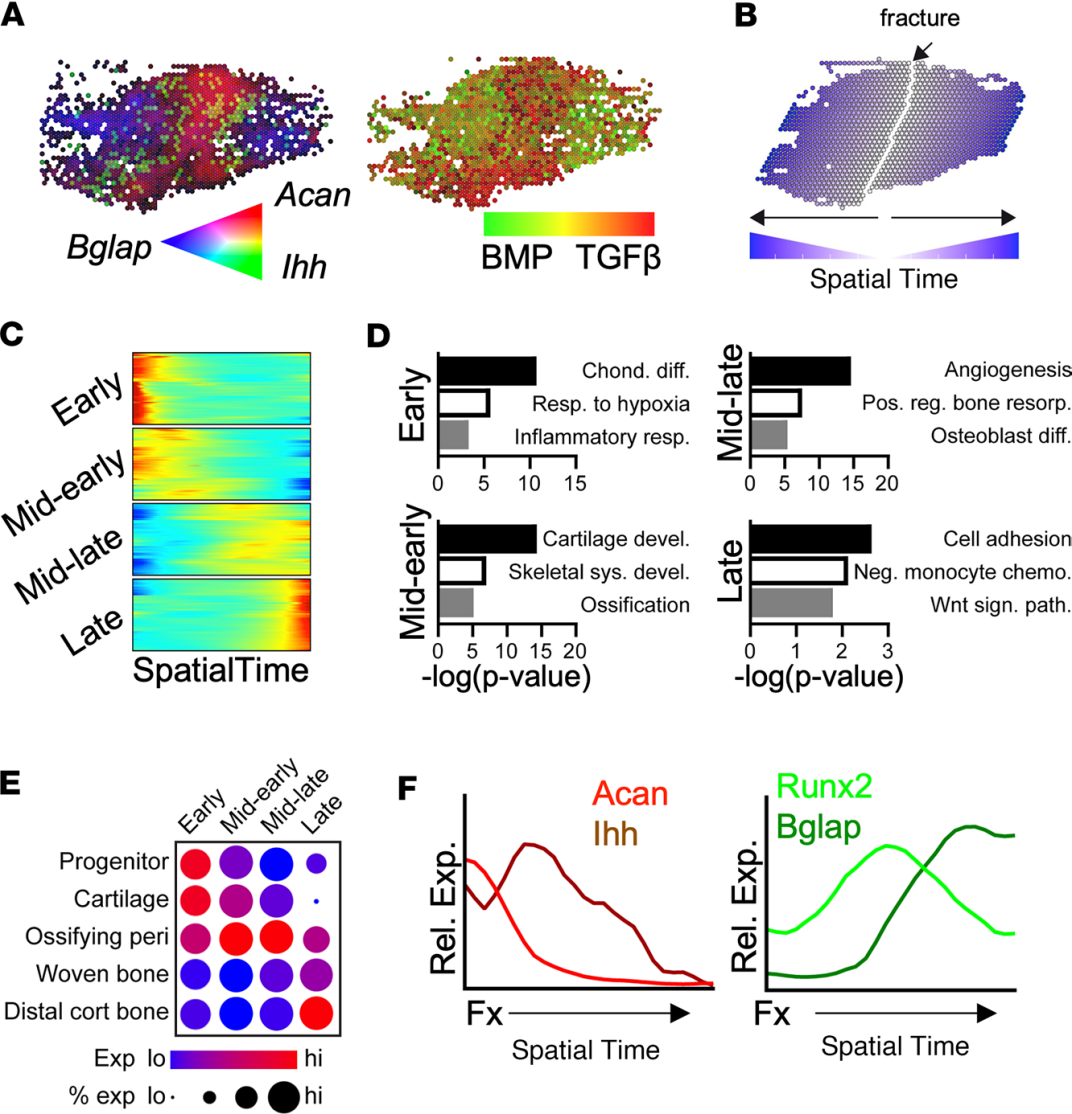
SpatialTime analysis of fracture healing. (**A**) Blended spatial feature plot of endochondral ossification markers (left), including cartilage (*Acan*), prehypertrophic chondrocytes (*Ihh*), and woven bone (*Bglap*), and TGF-β and BMP signaling (right). (**B**) Schematic of SpatialTime from the fracture plane within the callus. (**C**) Heatmap of gene expression across SpatialTime, delineating 4 classes with distinct dynamic expression patterns among skeletal spatial spots. (**D**) Enrichment analysis within each dynamic expression class. (**E**) Bubble plot depicting the relative expression level and percentage of spots with detectable expression of genes from each of the 4 classes in skeletal clusters from the control callus. (**F**) Relative expression of selected endochondral ossification markers across SpatialTime.

While cell clusters were localized to distinct regions within the fracture callus, we next sought to test whether spatial transcriptomics could delineate molecular signaling gradients associated with tissue diversity present throughout the control fracture callus. For this, we first localized genes associated with cartilage (*Acan*), prehypertrophic chondrocytes (*Ihh*), and woven bone (*Bglap*) within the control fracture callus, which showed a bidirectional gradient from the fracture plane (Figure 2A). These predicted cell populations corresponded to the differential expression of genes implicated in the TGF-β and BMP signaling pathways nearest and furthest from the fracture site, respectively, and in a manner consistent with the known role of these pathways in endochondral bone development (23) (Figure 2A). Based on this result, we hypothesized that molecular pathways important for fracture repair are expressed as signaling gradients defined by the relative distance from the fracture plane or by the distribution of spatially restricted tissue clusters within the callus and that these gradients are associated with distinct biologic processes required for normal bone regeneration after fracture. In support of this, we previously demonstrated the spatially restricted gradient expression of osteogenic molecular pathways within the embryonic mouse cranial suture (18), within the regenerating digit tip following amputation (17), and within the Achilles tendon following injury (24).

To assess transcripts that change along this proximal/distal axis without bias, we calculated SpatialTime (17, 18, 24, 25) for each skeletal cluster spatial spot, defined as the relative distance from the fracture plane (Figure 2B). Consistent with the histologic and spatial transcriptomic delineation of multiple endochondral cell types within the 10-day fracture callus (Figure 1, A–C), these reparative cell populations localized in a progressive manner from the fracture site, with cartilage and ossifying perichondrium localized nearest the fracture plane and newly formed woven bone and mature cortical bone furthest from the fracture site (Supplemental Figure 1A). This is further supported by the relative expression of endochondral gene sets throughout SpatialTime (Supplemental Figure 1B). While this linear axis analysis represents a simplistic view that all signaling gradients are derived from the fracture plane, these results suggest that tissue types within the callus are, for the most part, spatially organized along this axis and that SpatialTime analysis allows for concurrent detection of tissue-specific expression signatures throughout the callus.

Next, unbiased detection of all transcripts with SpatialTime-dependent gene expression (i.e., genes which showed variable expression along the proximal/distal axis) was conducted. These genes were grouped into 4 classes, including genes with high expression nearest the fracture site (early and midearly), genes with transiently increased expression later in SpatialTime (midlate), and genes with increased expression furthest from the fracture site (late) (Figure 2C). GSEA analysis identified significantly associated gene sets with each of the SpatialTime classes, transitioning from chondrogenic and ECM pathways near the fracture site to ossification and WNT signaling pathways at sites distant to the injury (Figure 2D). Supporting the concept that the SpatialTime axis includes tissue types and cell clusters broadly representing the various stages of endochondral healing, mapping of gene groups demonstrated enrichment of early and midearly genes in the progenitor and cartilage spatial clusters, while midlate and late genes were enriched in mineralizing tissues (Figure 2E). Consistent with this, SpatialTime analysis resolved the expression of genes indicative of the cartilage-to-bone transition during endochondral bone formation following fracture, including early- and late-stage chondrogenic (*Acan* and *Ihh*, respectively) and osteogenic (*Runx2* and *Bglap*, respectively) markers (Figure 2F). Taken together, our results demonstrate that endochondral expression gradients are spatially defined within the fracture callus, due, in part, to the spatially restricted heterogeneity of cell and tissue types within the fracture callus, supporting the tissue-specific roles that each morphogenetic pathway plays in the normal fracture healing process.

### Endochondral trajectory of spatial clusters during fracture healing.

Both the clustering and SpatialTime analyses illustrate the cellular heterogeneity (and associated gene expression signatures) within the fracture callus, reflecting the cartilage-to-bone transition indicative of endochondral ossification in vivo. While their relative proportions may change dramatically throughout the regenerative process after fracture, the 10-day postfracture callus consists of cells representing multiple stages of osteolineage commitment and differentiation. Therefore, we performed trajectory analysis to characterize the relationships among the diverse reparative tissue types present within the callus. While trajectory analysis infers a linear relationship between diverse cell states, as was previously demonstrated with mouse bone marrow cell populations (26), it does not imply a direct lineage fate. Despite this, trajectory analysis deconvolutes this inherent cellular heterogeneity to extract biologically relevant changes in gene expression potentially driving cellular transitions throughout endochondral repair (27).

**Figure 3:**
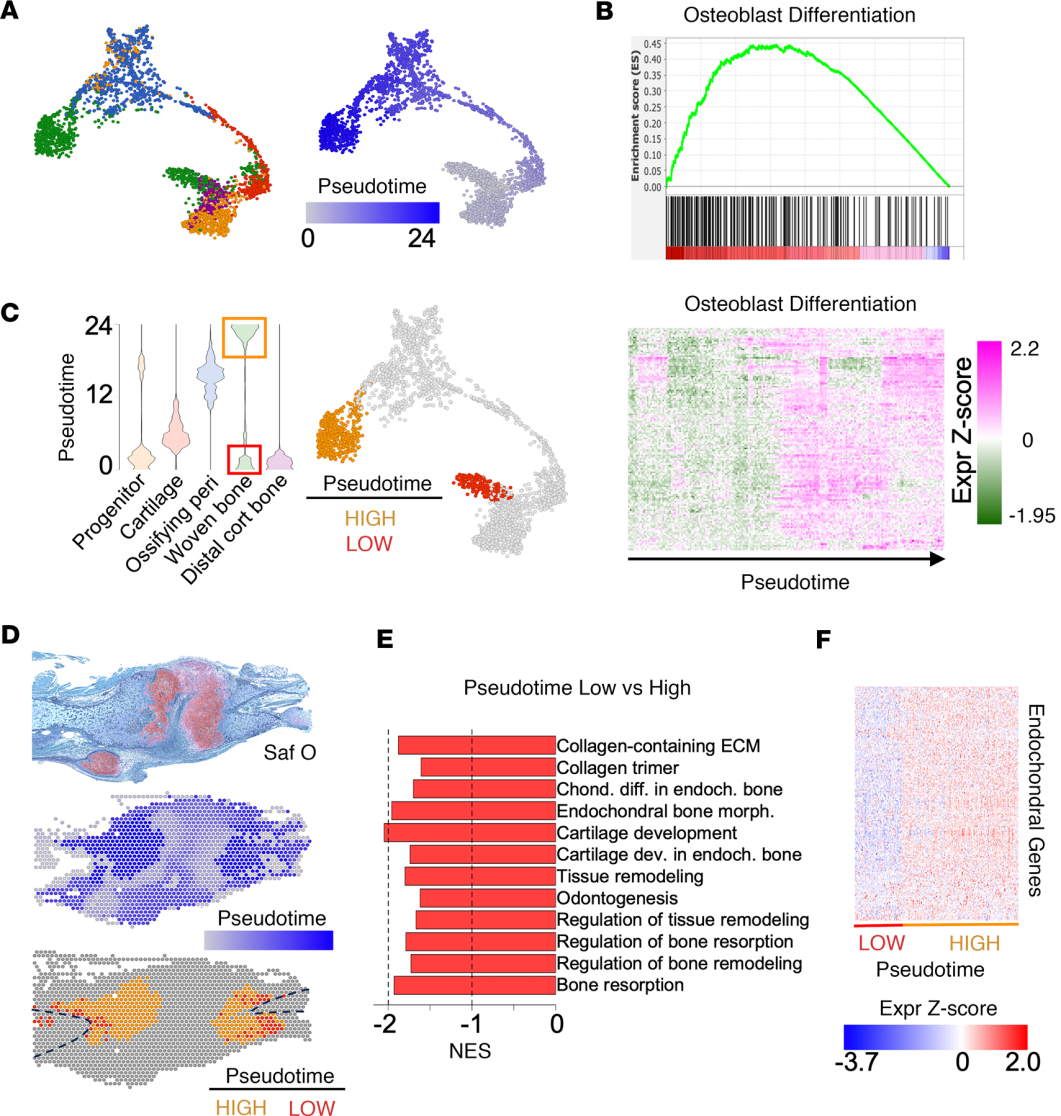
Pseudotime analysis of spatial clusters identifies an endochondral trajectory during fracture healing. (**A**) Trajectory analysis of spatial spots colored by skeletal cell cluster (left) or pseudotime (right). (**B**) Gene set enrichment (top) and heatmap of core genes (bottom) implicating osteoblast differentiation associated with trajectory pseudotime. (**C**) Violin plot of pseudotime distributions among skeletal cell clusters (left) and skeletal cluster trajectory indicating the relative position of pseudotime-high (orange) and pseudotime-low (red) woven bone spatial spots (right). (**D**) Safranin O histology (top), spatial spot pseudotime distribution (middle), and spatial distribution of pseudotime-high (orange) and pseudotime-low (red) woven bone spots (bottom) in a control fracture section. Dashed line indicates bone surface. (**E**) GSEA results demonstrating reduced expression of endochondral gene sets in the pseudotime-low compared with pseudotime-high woven bone spatial spots. (**F**) Heatmap of core genes (from **E**) in pseudotime-high and -low woven bone spatial spots.

Analysis of the control fracture detected a trajectory from the progenitor spatial clusters to a cartilage intermediate and then to the newly formed woven bone clusters (Figure 3A). Based on this trajectory, we calculated pseudotime, a numeric representation of the inferred linear trajectory (28, 29), for each spatial spot using the progenitor terminus as the root state (Figure 3A) and performed GSEA (Supplemental Table 8). Trajectory pseudotime was associated with increased expression of osteogenic genes, suggestive of endochondral ossification (Figure 3B). Interestingly, we observed a bimodal pseudotime distribution among the newly formed woven bone cluster in the control fracture (Figure 3C). One cluster was evident at the terminus of the trajectory deriving from the ossifying perichondrium (named here pseudotime-high). The second woven bone population derived directly from the progenitor population (named here pseudotime-low). The pseudotime-high and pseudotime-low populations were transcriptionally distinguishable (Supplemental Figure 2A), despite sharing the newly formed woven bone identity and being distinct from the other cell types within the callus. We hypothesized the pseudotime-low and -high populations may represent reparative bone formed through intramembranous and endochondral processes, respectively, which have both been implicated in fracture healing (30). Consistent with this, pseudotime-low cells localized further from the fracture plane (i.e., higher SpatialTime), compared with pseudotime-high cells, and along the cortical bone surfaces rather than adjacent to the fracture cartilage (Figure 3D and Supplemental Figure 2B). Furthermore, expression of *Col10a1*, a marker of chondrocyte hypertrophy indicative of endochondral ossification, was higher in the pseudotime-high cells localized near the fracture cartilage compared with the pseudotime-low cells (Supplemental Figure 2C). Finally, expression of endochondral osteogenesis gene sets was significantly reduced in the pseudotime-low population compared with the pseudotime-high population (Figure 3, E and F, and Supplemental Table 9). Taken together, spatial transcriptomic analysis of a control fracture callus identified transcriptionally and spatially unique forms of bone regenerate during the reparative process.

### Deficient endochondral bone formation in Nf1^Postn^ fracture defect.

**Figure 4:**
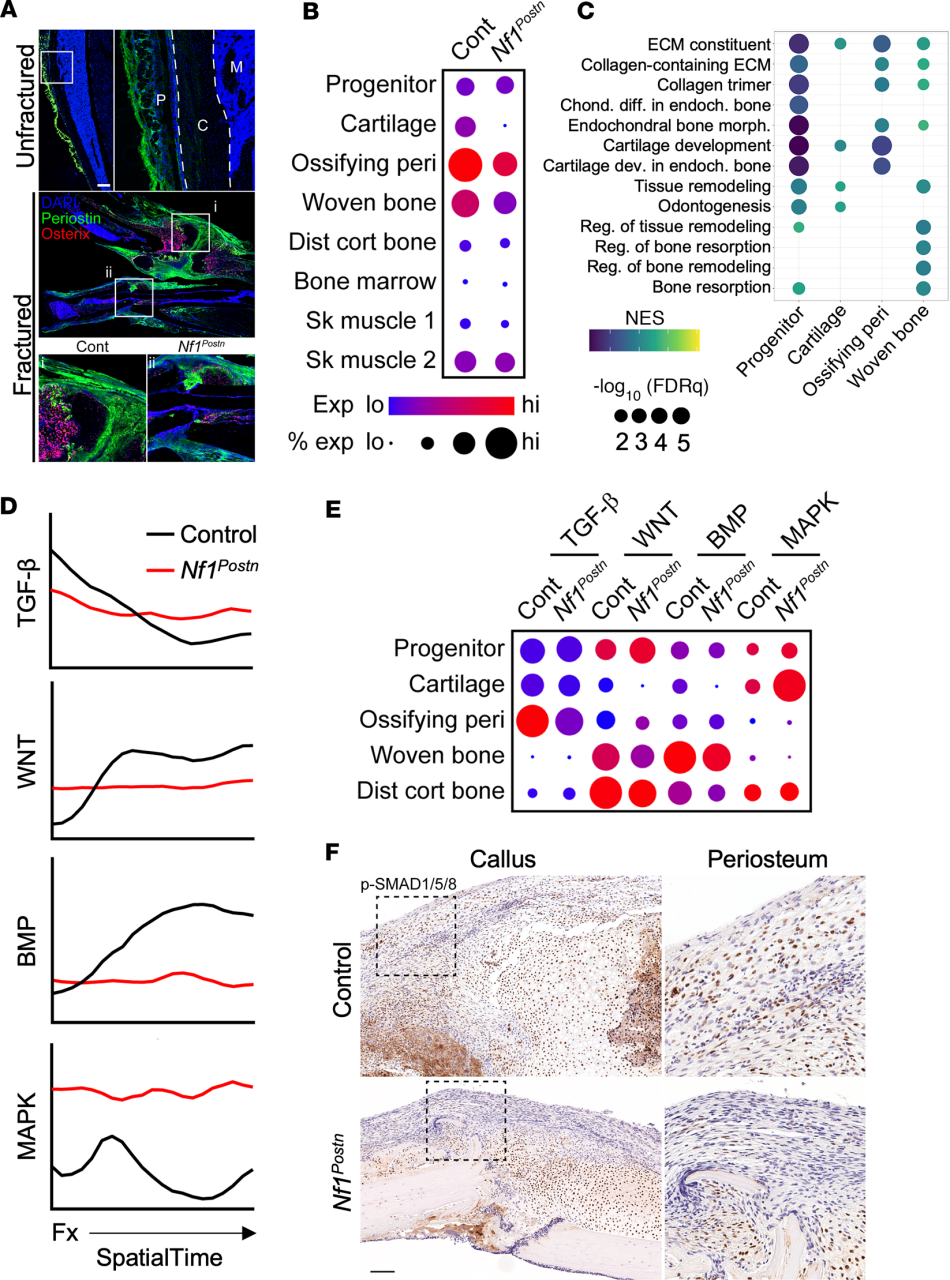
Spatial transcriptomic analysis of *Nf1*^Postn^ fracture pseudarthrosis reveals impaired endochondral bone formation. (**A**) Immunohistochemical localization of periostin (green) and the osteoprogenitor marker osterix (magenta) in uninjured mouse tibia (top) and spatial mouse fractures (bottom). P, periosteum; C, cortical bone; M, marrow. (**B**) Bubble plot of *Nf1* expression and percentage of expressing spots across cell clusters of the control and *Nf1*^Postn^ callus. (**C**) Bubble plot depicting cluster-associated gene sets with significantly reduced expression in *Nf1*^Postn^ fracture sections compared with control sections. (**D**) Relative expression of selected bone morphogenetic and MAPK pathways in the control and *Nf1*^Postn^ fractures across SpatialTime. Fx, fracture. (**E**) Bubble plot expression of selected bone morphogenetic and MAPK pathways in the control and *Nf1*^Postn^ fractures across skeletal cell clusters. (**F**) Immunohistochemical localization of phospho-SMAD1/5/8 in the control (top) and *Nf1*^Postn^ (bottom) fracture calluses. Scale bar: 100 μm.

Having established the utility of spatial transcriptomics in defining the normal transcriptional landscape within a control fracture callus, we next sought to evaluate the molecular dysregulation associated with delayed fracture healing in a preclinical *Nf1* GEMM. Conditional deletion of *Nf1* in the bone periosteum of *Nf1*^Postn^ mice results in impaired endochondral skeletal development and delayed fracture healing (14). Prior to fracture, periostin protein was restricted to the bone periosteum but becomes more broadly expressed within the fracture callus after injury, reflecting the periosteal response to injury 10 days after fracture (Figure 4A). Spatial transcriptomic data from the *Nf1*^Postn^ fracture were analyzed and harmonized with the control callus data to avoid bias in cluster identification and characterization. The number of osteochondral-committed spatial spots was notably reduced within the *Nf1*^Postn^ fracture compared with control fracture (Supplemental Figure 3A). Assessment of *Nf1* expression within each of the identified spatial clusters showed notable reductions in the cartilage, ossifying perichondrium, and woven bone within the *Nf1*^Postn^ callus (Figure 4B). Expression of spatial cluster-associated endochondral gene sets detected in the control callus (Figure 1D), such as ECM formation, cartilage development, and bone formation and remodeling, was significantly reduced in tissue-matched skeletal spatial clusters from *Nf1*^Postn^ fracture sections compared with control fractures (Figure 4C, Supplemental Figure 3B, and Supplemental Tables 10–13). Consistent with this molecular evidence for impaired endochondral ossification following fracture, histologic analyses detected minimal cartilage formation and robust collagen deposition at the *Nf1*^Postn^ fracture site (Supplemental Figure 3, C and D) compared with the control site (Figure 3D). Moreover, expression of endochondral gene sets was similarly reduced in the *Nf1*^Postn^ cortical bone further from the fracture plane compared with control cortical bone spatial spots (Supplemental Figure 3E and Supplemental Table 14), further demonstrating impaired endochondral bone formation in *Nf1*^Postn^ mice.

Previous studies have implicated impaired BMP, WNT, and TGF-β pathway signaling in osteogenic differentiation defects associated with loss of *Nf1* (7, 12, 31). As has been demonstrated with multiple GEMMs, osteogenic differentiation of *Nf1*-deficient progenitor cells was reduced and not rescued with BMP2 (12, 32). Similarly, WNT signaling and osteogenesis were inhibited in bone marrow stromal cells cultured from adult *LepR*-cre;*Nf1*^fl/–^ mice, and this was not rescued by treating cells with the potent WNT agonist osteolectin (7). Using SpatialTime analysis, we next tested the spatial activation of these morphogenetic pathways previously associated with skeletal development and fracture repair in the control and *Nf1*^Postn^ calluses. Control fractures showed peak activation of TGF-β, WNT, and BMP signaling throughout SpatialTime corresponding to the chondrogenic, osteochondral junction, and osteogenic regions of the callus, respectively (Figure 4D). In comparison, analysis of the *Nf1*^Postn^ callus revealed that, similar to the control callus though to a slightly lesser extent, TGF-β pathway genes were expressed highest near the fracture plane, consistent with in vitro analyses of *Nf1*-deficient bone marrow stromal cells (33). In contrast, little expression of WNT or BMP pathway genes was observed throughout *Nf1*^Postn^ fracture SpatialTime (Figure 4D). Reduced WNT and BMP pathway expression correlated with increased MAPK pathway activation in the *Nf1*^Postn^ fracture, consistent with loss of *Nf1* (Figure 4D). Similar results were observed when pathway activation was assessed in each spatial cluster (Figure 4E). Confirming our spatial analyses, robust phospho-SMAD1/5/8 staining, indicative of BMP pathway activation, was evident throughout the control callus, including along the bony ends of the fractured tibia and the periosteum (Figure 4F). In contrast, the *Nf1*^Postn^ fracture callus showed less activated SMAD staining along the bony ends, particularly within the periosteum (Figure 4F and Supplemental Figure 3F). Together, these results suggest that, while TGF-β activation, known to drive chondrogenesis, was only modestly deficient in the *Nf1*^Postn^ fracture callus, the pathogenesis of healing defects in mutant mice is primarily driven by impaired WNT and BMP signaling needed for the transition to endochondral healing. Thus, spatial transcriptomics provides unbiased in situ evidence implicating these pathways, consistent with prior in vitro results from other *Nf1* GEMMs (7, 12).

### Transcriptome profiling identifies MAPK-associated osteogenic dysregulation in patient fracture-derived primary cells.

**Figure 5:**
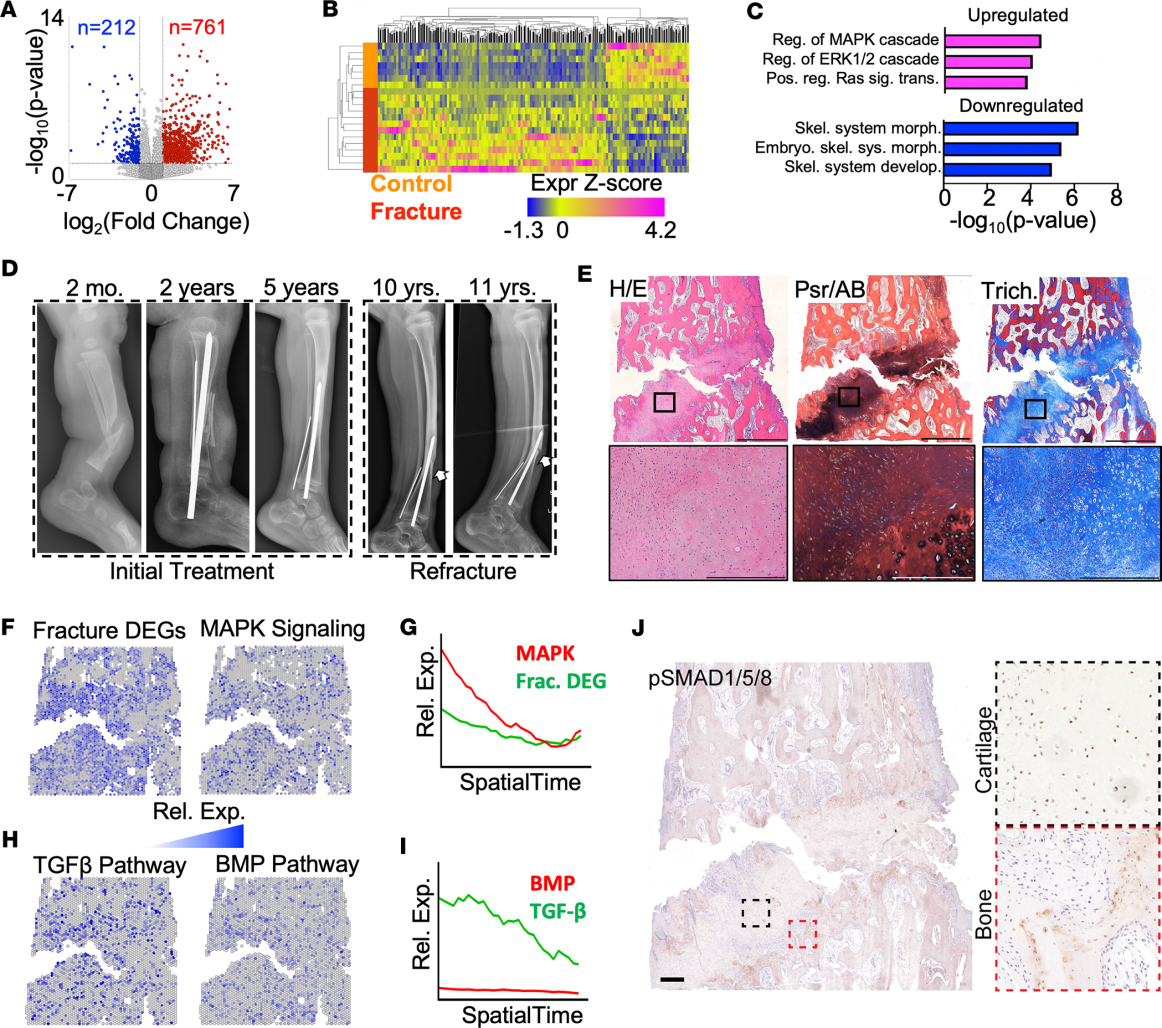
Spatial transcriptomic analysis of patient-derived fracture pseudarthrosis tissue reveals a deficiency in BMP signaling. (**A** and **B**) Volcano plot (**A**) and heatmap (**B**) showing DEGs between primary cells derived from patients with NF1 fracture pseudarthrosis and primary cells cultured from control bones from patients with NF1. (**C**) GSEA results demonstrating enrichment of pathways for DEGs upregulated (magenta) and downregulated (blue) in primary cells derived from patients with NF1 fracture compared with primary cells from patients with NF1 acting as controls. (**D**) Longitudinal radiography of a patient with NF1 fracture pseudarthrosis demonstrating initial fracture at birth and subsequent refracture. Arrows indicate location of the refracture. (**E**) Histologic evaluation of patient fracture pseudarthrosis, including H&E (left), picrosirius red/alcian blue (middle), and Masson’s trichrome (right) staining. (**F** and **G**) Spatial expression (**F**) and relative SpatialTime expression (**G**) of DEGs (Frac. DEGs; from **A**) and MAPK pathway genes. (**H** and **I**) Spatial expression (**H**) and relative SpatialTime expression (**I**) of TGF-β and BMP pathway genes. (**J**) Immunohistochemical localization of phospho-SMAD1/5/8 in the human NF1 pseudarthrosis specimen. Scale bar: 100 μm.

To determine the underlying mechanism driving human pseudoarthrosis, we first performed transcriptome analyses of primary control cells (i.e., *NF1*^+/–^) cultured from unaffected bone (*N* = 7 patients) and primary mosaic (i.e., mixed *NF1*^+/–^ and *NF1*^–/–^) cells cultured from pseudarthrosis fractures (*N* = 13 patients) from patients with clinical diagnoses of NF1. Transcriptome analysis detected 761 and 212 differentially expressed genes (DEGs) with significantly increased or decreased expression in fracture-derived primary cells compared with control cells, respectively (Figure 5, A and B, and Supplemental Table 15). Using GSEA, genes with increased expression in fracture-derived primary cells were enriched in the RAS/MEK/ERK (MAPK) signaling pathway, consistent with loss of *NF1* (Figure 5C and Supplemental Table 16). Moreover, genes with reduced expression in fracture-derived primary cells were enriched in pathways implicated in skeletal development (Figure 5C and Supplemental Table 17). These results suggest that MAPK-dependent suppression of osteogenic genes is associated with NF1 fracture pseudarthroses. However, analyses of patient-derived primary cells in culture does not provide spatial context within the fracture pseudarthrosis.

### Spatial transcriptomics identifies impaired BMP signaling in human NF1 fracture pseudarthrosis.

We next sought to delineate the molecular landscape within a pseudarthrosis specimen from a patient with NF1. Spatial transcriptomic analyses were performed on a pseudarthrosis fracture resected en bloc from a patient with NF1 during surgery performed as standard of care. The patient initially presented at 2 months of age with pseudarthrosis following fracture presumed to have occurred in utero (Figure 5D). The initial fracture was treated with an intramedullary rod, bone graft, and rhBMP2 at 2 years of age. The fracture was radiographically healed, and the dysplastic tibia grew with the support of a brace without further complication. At 10 years of age, the patient complained of pain, and a fracture of the anterior cortex was observed by radiograph and treated with bracing. One year later, the fracture remained unhealed and was treated surgically, during which the pseudarthrosis site was resected for spatial analysis.

The resected pseudarthrosis was histologically demarcated with a fibrocartilage fracture plane flanked by mineralized bone (Figure 5E). The fracture plane consisted mostly of collagen-rich fibrous tissue, with sporadically detectable regions of chondrocytes (detected by picrosirius red/alcian blue) (Figure 5E). Spatial transcriptomic analysis was performed on 2 sections of the patient pseudarthrosis tissue. Dimensionality reduction with principal component analysis followed by graph-based clustering identified 4 distinct clusters (Supplemental Figure 4A). As expected, cluster 1 (green) localized along the fracture plane and expressed high levels of the fibrillar collagen *COL11A1* and the cartilage marker gene *HAPLN1* (Supplemental Figure 4, A and B). To further identify the 4 unique spatial clusters within our clinical patient sample, differential gene expression analysis was performed and results were compared with mouse spatial clusters (Supplemental Table 18). Cross-species mapping was conducted to determine which human spatial cluster most closely resembled each mouse fracture spatial cluster (Supplemental Figure 4C) or, conversely, which mouse cluster most closely resembled each human spatial cluster (Supplemental Figure 4D). These data, combined with morphological findings, suggest that human spatial cluster 1 (hCluster 1) likely represents a form of fibrocartilage, and hCluster 3 resembles more osteogenic tissue, while hClusters 2 and 4 likely represent mixed populations of osteogenic and hematopoietic cell populations.

To spatially resolve the molecular dysregulation associated with NF1 pseudarthrosis, we evaluated the spatial distribution and relative expression throughout SpatialTime (Supplemental Figure 4E) of upregulated DEGs identified by RNA-Seq (Figure 5, A and B). Upregulated fracture-associated DEGs were more highly expressed at the fibrocartilage-osseus junction within the pseudarthrosis tissue, as were MAPK pathway–associated genes (Figure 5, F and G). We next localized the expression of TGF-β and BMP pathway-associated genes within the pseudarthrosis tissue. Consistent with SpatialTime results from the mouse control fracture (Figure 4D), TGF-β pathway genes were expressed within the fibrocartilage tissue adjacent to the fracture plane and declined further from the fracture site (Figure 5, H and I). In contrast, expression of BMP pathway genes was poorly detected within the pseudarthrosis tissue (Figure 5, H and I). Though human healthy, normally healing fracture tissue is not typically available for comparison, our results do suggest that impaired BMP pathway activation is conserved in both the human and mouse *NF1*-deficient fractures. To validate these results, we evaluated phospho-SMAD1/5/8 staining within the human fracture tissue. Consistent with the *Nf1*^Postn^ fracture callus, phospho-SMAD1/5/8 was evident in the mineralized bone of the patient fracture; however, little BMP activation was evident within the fracture cartilage or the transition zone (Figure 5J). These results suggest a lack of BMP pathway activation contributes to NF1-associated fracture pseudarthrosis in patients.

Finally, we sought to further evaluate the impaired BMP response as a potential mechanism for NF1-associated pseudarthrosis by integrating both the human and mouse data sets. We hypothesized that genes with significantly reduced expression in human fracture-derived primary cells, which were associated with skeletal gene sets (Figure 5C), may colocalize within the BMP-enriched signaling gradient during normal fracture healing in mice. In support of our hypothesis, the expression of skeletal DEGs downregulated in human pseudarthrosis-derived primary cells colocalized within the BMP signaling gradient of the control mouse fracture callus (Supplemental Figure 4F). Taken together, cross-species comparative spatial transcriptomic analyses of fracture tissue implicate impaired BMP signaling in the pathogenesis of NF1-associated fracture pseudarthrosis.

## Discussion

Spatial transcriptomic analyses of pathologic tissues have the potential to unravel molecular mechanisms and biomarkers of disease within their native microenvironment. However, the application of spatial transcriptomics to skeletal tissues, either under normal physiologic or disease states, has been limited due, in part, to the difficulties in accessing cell populations embedded in mineralized tissue while also maintaining RNA integrity. Here, we demonstrate the cross-species application of spatial transcriptomics to elucidate molecular mechanisms associated with a skeletal disease.

Spatial transcriptomic analysis of mouse fractures revealed distinct skeletal, muscle, and bone marrow clusters within or adjacent to the fracture callus. Expression of gene sets required for endochondral bone formation, such as cartilage formation, ECM formation, and ossification, were associated with different skeletal clusters, reflecting the diverse cell types involved in the normal endochondral reparative process after fracture. Consistent with the localization of these tissues within the callus, the expression of key morphogenetic pathways was spatially restricted within the fracture callus. In a similar manner, we performed spatial transcriptomic analyses using a preclinical model of delayed fracture healing associated with loss of periosteal *Nf1* expression. As previously reported (14), fracture healing was delayed in *Nf1*^Postn^ mice compared with control mice. Consistent with the lack of cartilage at the fracture site, expression of molecular pathways involved in endochondral bone formation was significantly reduced in all skeletal spatial clusters in *Nf1*^Postn^ sections, including in cortical bone distant from the fracture site. Like that in control fracture, spatial expression of TGF-β pathway genes was highest near the fracture plane in the *Nf1*^Postn^ fracture. In contrast, spatial transcriptomic analyses demonstrated a lack of WNT and BMP pathway activation within the *Nf1*^Postn^ fracture, potentially contributing to the delayed fracture healing in these mice. Interestingly, our data suggest that MAPK overactivation in *Nf1*^Postn^ mice was most prominent near the cartilage-like region of the fracture callus, with histological evidence confirming a dramatically impaired ability to undergo endochondral healing. Previous in vitro studies have shown a critical role for MAPK signaling during TGF-β–induced chondrogenesis, where treatment with the MEK inhibitor PD98059 or siRNA-mediated knockdown of p38 showed increased expression of chondrogenic genes and glycosaminoglycan production (34, 35). These results are consistent with our human specimen analysis, which showed impaired cartilage formation within the fracture site. Further work is needed to understand the cartilage-specific effects of MAPK hyperactivation observed in *NF1* deficiency and how this may contribute to pseudarthroses formation.

Spatial transcriptomics also allows for the analysis of signaling gradients to contextualize the molecular and cellular heterogeneity within the fracture callus. Our results demonstrate the feasibility of delineating the molecular and tissue-specific heterogeneity inherent in complex regenerative processes, such as fracture healing, to reconstruct phase transitions representing endochondral bone formation in vivo, and to spatially delineate molecular mechanisms of disease. More in-depth analyses in the future may help to establish signaling gradients established along other dimensional axes (e.g., dorsal/ventral or anterior/posterior) or derived from other major signaling hubs such as the outer periosteum, cortical bone surface, or surrounding muscle. Additionally, inclusion of additional time points representing different stages of the healing process will further resolve the role that such spatial diversity of cell types plays in coordinating healing and remodeling after fracture. Finally, spatial transcriptomic analyses of additional mouse models may uncover additional pathways contributing to normal or delayed fracture healing. As prior genetic and histologic evaluations of human specimens have implicated the periosteum in the etiology of this disease, we analyzed fractures from *Nf1*^Postn^ mice; however, analyses of patient specimens will be required for comparison to GEMMs to ultimately uncover the molecular basis of disease with this technique.

To correlate preclinical spatial transcriptome results to the human disease, we similarly analyzed a fracture pseudarthrosis specimen from a patient with NF1. Within the fracture tissue, expression of MAPK pathway genes was highest at the fibrocartilage-osseus transition zone nearest the fracture line. As expected, expression of TGF-β pathway genes was highest near the fracture and declined further from the fracture plane. In contrast, expression of BMP pathway genes was minimal throughout the fracture section. We hypothesize that this dramatic reduction in BMP signaling during NF1 fracture repair leads to pseudarthrosis. Consistent with this, genes with the most reduced expression in pseudarthrosis-derived primary cells localize to the region of the control mouse fracture callus enriched for BMP pathway expression. Our integrated spatial transcriptome analyses implicate a loss of BMP signaling gradient during fracture repair in the pathogenesis of NF1 pseudarthrosis.

Fracture pseudarthrosis in children with NF1 is commonly treated surgically with resection of the fibrous callus, placement of intramedullary fixation, and addition of bone graft and rhBMP2 (2). The clinical utility of off-label rhBMP2 is controversial, with no clear reproducible benefit in limiting refracture rates (4, 36). A recent multicenter retrospective study found lower rates of union when rhBMP2 was utilized in patients with NF1 (37). Our integrated spatial transcriptome analyses of preclinical and patient fracture tissue provide in situ molecular evidence for impaired BMP pathway activation following fracture in patients with NF1. In support of this, MAPK activation was previously shown to inhibit BMP/Smad signaling, and the osteogenic response of *Nf1*-deficient osteochondroprogenitors to BMP2 treatment was rescued with the addition of a MEK inhibitor (12, 38). Taken together, the lack of BMP pathway response during fracture healing and the activated MAPK signaling within NF1 pseudarthrosis tissue may suggest that the combination of rhBMP2 and a MEK inhibitor may improve fracture healing in patients with NF1.

Application of spatial transcriptomic analyses has been mostly limited to noncalcified tissues, such as brain, heart, kidney, and various cancers (39). While a few studies have successfully applied this approach to skeletal structures, including neonatal cranial sutures (18), amputated digit tip (17), and, most recently, in mouse femurs (25), analyses of preclinical models of skeletal disease have been limited, and no study has evaluated pathologic patient-derived skeletal tissues. Our results demonstrate the feasibility of investigating mechanisms of disease in skeletal tissues, suggesting such an approach may elucidate the molecular basis of other skeletal pathologies with spatial context. Spatial transcriptomic analyses of skeletal pathologies may be particularly suited to archival FFPE samples, including for both preclinical and clinical specimens. The use of fixed skeletal tissues allows for decalcification of the specimen and liberation of RNA molecules, though this remains a key limitation. The specimens utilized in this study underwent standard decalcification with EDTA with daily monitoring by radiography. Decalcification was halted when mineralized tissue was absent. Because the decalcification procedure negatively affects RNA quality, alternative strategies for decalcification may improve the performance of spatial transcriptome detection in fixed skeletal tissues.

It remains possible, given the limitations in spatial resolution of this approach, that observed differences in gene expression, such as were noted here between pseudotime-high endochondral spots and pseudotime-low intramembranous spots, could be influenced by the proximity of cells to other tissues. Further analyses, for example, utilizing different injury models more reliant on intramembranous healing, will be required to elucidate the molecular and spatial trajectories of differing endochondral and intramembranous ossification fronts during fracture healing. However, our data suggest the ability of spatial transcriptomics to interrogate these unique processes. Moreover, as spatial transcriptomics continues to advance toward single-cell resolution, delineation of individual cell populations within distinct microenvironments will further elucidate the cellular heterogeneity of different skeletal niches and further resolve the cell type–specific molecular basis of skeletal, and possibly somatic, diseases. Beyond characterizing cellular heterogeneity, this approach may also enable analyses of skeletal pathologies that are secondary to bone metastases or infiltrating neoplasia, such as occurs with NF1 plexiform neurofibromas (40), or with other skeletal tumors (41). Such studies have potential to elucidate the molecular crosstalk at the bone-tumor interface. Spatial transcriptomics enables unbiased molecular analysis of preclinical and clinical disease specimens, and results from our study reveal opportunities to investigate the molecular basis of skeletal disease pathologies.

## Methods

### Sex as a biologic variable.

Female mice were utilized for spatial transcriptomic analyses. Findings are expected to be relevant to both sexes, as previously reported for *Nf1*^Postn^ mice (14). Both male and female participants were included for patient primary cell transcriptome experiments. Race and ethnicity were not selected, and inclusion was determined based on the clinic census.

### Animal procedures.

As previously described, *Nf1*^Postn^ mice were generated by crossing *Postn*-cre^+^;*Nf1*^fl/fl^ mice with *Nf1*^+/–^ mice (14). Two-month-old female mice were anesthetized with isoflurane, and open tibia osteotomies were introduced using a bone blade into the distal tibia at or below the tibia/fibula junction. Tibiae were stabilized with an intramedullary 0.3 mm diameter pin, and the surgical site was closed with nonabsorbable sutures. Fractures and pin stabilization were confirmed by radiography 24 hours after surgery. Visium Gene Expression System (10X Genomics) experiments were performed using *Postn*-cre^-^;*Nf1*^+/fl^ (control) and *Nf1*^Postn^ gender-matched littermate mice.

### Mouse tissue collection, histology, and immunofluorescence.

Fracture calluses were harvested 10 days after fracture. The intramedullary pin was extracted and excess tissue removed from around the bone and callus site. The distal ends of the bone were cut to expose the intramedullary canal, and the callus was fixed in 10% formalin overnight at 4°C. Decalcification was performed in 14% EDTA at 4°C and monitored by daily radiography until there was no evidence of calcified tissue (~3 days). Following decalcification, calluses were rinsed and submitted to the University of Texas Southwestern Medical Center Histo-Pathology Core. Without delay, samples were dehydrated with ethanol, cleared with xylene, infiltrated with wax, and embedded longitudinally. Paired samples of fracture calluses from both control and *Nf1*^Postn^ mice were embedded in the same block with differing geometry such to discern control from mutant. Blocks were faced to near center of callus where full-face sections comprising 50 μm block depth were collected for RNA integrity analysis. RNA quality was evaluated by RNA TapeStation analysis (Agilent) following extraction using the RNeasy FFPE RNA extraction kit (Qiagen). The DV200 value (recommended >50%), representing the percentage of transcripts at least 200 nucleotides in length, was utilized to confirm suitability in downstream Visium analysis. Blocks were sectioned at 5 μm, and serial sections were applied across slides for H&E, Safranin O, and Visium.

For immunofluorescence, paraffin sections were deparaffinized with 3 5-minute washes in xylenes, followed by 100% ethanol, followed by reducing concentrations of ethanol, and ending in distilled water. Sections were then permeabilized for 10 minutes with 0.05% NP40 and 2.5% DMSO in PBS and then washed in PBS and blocked in PBS with 5% normal donkey serum (Jackson Immunoresearch) for 1 hour at room temperature. Slides were then stained overnight at 4°C in PBS containing 5% normal donkey serum with goat anti-periostin (1:100, AF2955, R&D Systems) and rabbit anti-osterix (1:200, ab22552, Abcam) antibodies. Slides were then stained for 2 hours at 4°C in PBS containing 5% normal donkey serum with donkey anti-goat Alexa Fluor 488 (1:250, Jackson Immunoresearch) and donkey anti-rabbit CF-555 (1:500, Biotium) antibodies. Sections were washed with PBS 3 times and then counterstained with DAPI to stain nuclei before mounting. Slides were mounted with Prolong Gold anti-fade reagent (Invitrogen). Images were acquired with a Zeiss LSM880 confocal microscope using the tiling function, and tiled images were then stitched together using Zen software (Zeiss) to create a composite image of the tissue. Confocal images were processed using Fiji (ImageJ) and Photoshop (Adobe Systems).

### Patient tissue collection, processing, and histology.

Pseudarthrosis tissue was resected en bloc during resection performed as standard of care. Tissue was fixed in 10% formalin overnight at 4°C. Decalcification was performed in 14% EDTA at 4°C and monitored by daily radiography until there was no evidence of calcified tissue (~2 weeks). Following decalcification, calluses were rinsed, submitted to the University of Texas Southwestern Medical Center Histo-Pathology Core, and processed for Visium analysis similarly as were the mouse fracture specimens.

For phospho-SMAD1/5/8 staining, deparaffinized sections were incubated using Proteolytic Enzyme digestion (0.25% Trypsin in 1 mM EDTA) at 37°C for 3 minutes and washed with distilled water. Endogenous peroxidase activity was deactivated using 3% H_2_0_2_ in methanol for 10 minutes at room temperature, rinsed in distilled water, and transferred to PBS with 0.05% Tween20 (PBST) pH 7.4 (MilliporeSigma). Sections were blocked with 10 % normal goat serum (Abcam, ab7481) for 1 hour at room temperature and incubated overnight at 4°C with the rabbit anti-phospho-Smad1/5/8 antibody (MilliporeSigma, AB3848-1, 1:100). Sections were washed with PBST and incubated with goat anti-rabbit IgG antibody conjugated to HRP (Millipore, AP187P; 1:500) for 1.5 hours and washed with PBST. Slides were treated with diaminobenzene solution (Dako, K3468) for 10 minutes and washed in distilled water. Sections were counterstained with Mayer’s hematoxylin (MilliporeSigma, MHS80-2.5L) for 2 minutes, washed, dehydrated in ascending grade of alcohol (70%, 95%, 100%), cleared in Xylene, and mounted in Cytoseal XYL (Thermo Scientific, 8312-4). Negative controls were run in parallel using rabbit IgG negative control (Abcam, ab27478). All sections were examined under Olympus BX63 microscope with DP73 camera. Digital images were obtained using Olympus cellSens imaging software.

### Visium processing and sequencing.

Spatial experiments were carried out in accordance with manufacturer’s guidelines. Briefly, sections were captured on a Visium slide, which contains poly-T sequences lining the slide surface. These poly-T probes additionally contained a unique spatial barcode that is used downstream to determine the 2D position for each piece of sequenced library. Samples were subjected to a modified H&E staining protocol and imaged. Next, samples were probed using a library gene set consisting of approximately 20,000 pair sequences. Following hybridization of gene probes to samples and removal of unbound probe fractions, hybridized probes were liberated and allowed to anneal to the spatial slide surface. On-slide DNA synthesis, clean up, and indexing were then carried out. Successful library construction was confirmed by bioanalyzer prior to sequencing. Samples were sequenced in a 2 × 150 bp configuration on a NovaSeq Illumina sequencer.

### Visium sequence analysis.

Most analyses were performed using Partek Flow software. Reads were demultiplexed and aligned to the mouse (mm39) or human (hg38) reference sequence using the *SpaceRanger* pipeline. Genes expressed in less that 0.3% of spots were excluded from further analyses. Expression values were normalized [log_2_(counts per million +1)]. Compared with the mouse fracture callus, fewer genes were detected in the patient specimen, likely due to the aggressive decalcification required for such a relatively larger piece of human bone (Supplemental Table 1). UMAP plotting and graph-based clustering were performed using the top 20 principal components. Differential gene expression analyses were performed using ANOVA and the Partek Gene Specific Analysis. Trajectory analysis was performed with Monocle software (28, 29, 42) using the top 2,000 genes with the greatest expression variance in the control fracture. Enrichment analyses were performed using the Gene Set Enrichment Analysis software (v4.2.3) (43, 44).

SpatialTime analysis (17, 18) was conducted by assigning spatial spots a value indicative of their distance to the fracture plane. DEGs changing as a factor of SpatialTime in the mouse fracture were calculated using Monocle, and pathway analysis of SpatialTime gene clusters was carried out using DAVID (45). For overall pathway activation, the *AddModuleScore* function of *Seurat* was employed, using established gene lists derived from KEGG pathways.

### Patient primary cell culture and transcriptome analysis.

Patient-derived primary cells were cultured from surgically resected tissues, including fracture pseudarthroses and excess autologous bone graft as previously described (6, 7). Primary cells were cultured in minimum essential media alpha (Thermo Fisher Scientific) supplemented with 10% fetal bovine serum (Thermo Fisher Scientific) and 1% antibiotic. Cells were harvested in RLT plus lysis buffer (Qiagen), and RNA was extracted using the RNeasy plus minikit (Qiagen). RNA quality was evaluated by Tapestation (Agilent) followed by library preparation. Following ribosomal RNA depletion, Illumina sequencing libraries were produced using the Illumina TruSeq library preparation kit. Libraries were sequenced using either 100 bp or 150 bp paired-end read configuration.

All transcriptome sequence analyses were conducted in Partek Flow software. Sequence reads were mapped to the human reference genome (hg38) using STAR software (46). Following duplicate read removal, expression was quantified relative to Ensembl protein-coding transcripts and normalized using the median ratio method using DESeq2 (47). DEGs were detected using DESeq2. Heatmap visualizations and GSEA analyses were conducted in Partek.

### Statistics.

For spatial transcriptomics, differential gene expression analyses were performed using 1-way ANOVA and the Partek Gene Specific Analysis. DEGs detected by transcriptome profiling of patient-derived primary cells were detected using DESeq2, and significance was determined by *P* value. For Supplemental Figure 3F, statistically significant differences were determined by 2-sided Student’s *t* test, and data represent mean ± SEM. A *P* value of less than 0.05 as considered significant.

### Study approval.

All animal procedures were approved by the Institutional Animal Care and Use Committee of the University of Texas Southwestern Medical Center. All patient-derived samples were collected as part of a research study approved by the Institutional Review Board of University of Texas Southwestern Medical Center following written informed consent.

### Data availability.

Mouse Visium fracture data are available in the NCBI’s GEO database (GSE218046). Values for all data points in graphs are reported in the Supporting Data Values file.

## Author contributions

JJR, CAW, and RJT designed the study. JJR, YHK, and RJT analyzed the data. CJ, JMS, NP, IO, RC, and ECJ conducted experiments. MW, DAP, and SJC provided reagents. JJR and RJT wrote the manuscript.

## Supplementary Material

Supplemental data

Supplemental tables 1-18

Supporting data values

## Version 1 07/11/2024

In-Press Preview

## Version 2 08/22/2024

Electronic publication
