# Supplementary material for: Spatial transcriptomics implicates impaired BMP signaling in NF1 fracture pseudarthrosis in murine and patient tissues
Source: JCI Insight. 2024 Jul 11;9(16):e176802. doi: 10.1172/jci.insight.176802 (PMC11343587; doi:10.1172/jci.insight.176802)
Supplement: Supplemental data [file jciinsight-9-176802-s149.pdf]

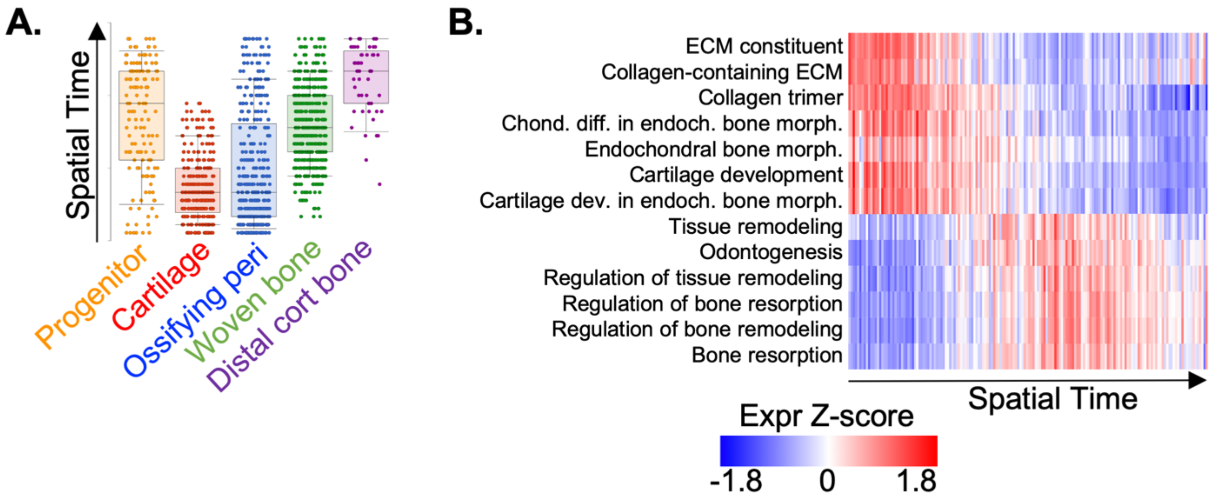

Supplemental Figure 1. **SpatialTime analysis of cluster-associated molecular pathways**.

(A) Box plot distribution of SpatialTime for each skeletal spatial cluster. **(B)** Heatmap expression of cluster-associated gene sets throughout SpatialTime.

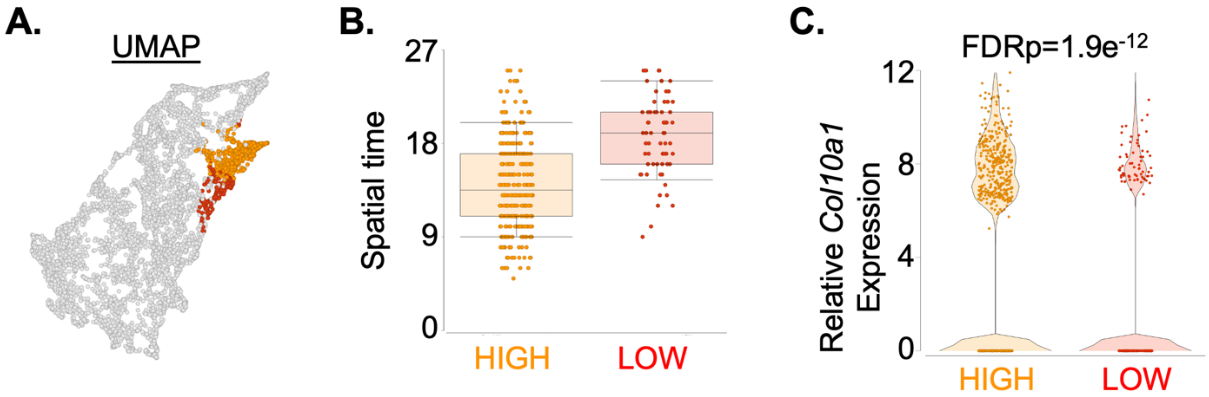

Supplemental Figure 2. **Bimodal pseudotime distribution of the woven bone spatial cluster**. **(A)** UMAP plot of all spatial cells highlighting the segregation of pseudotime-high and -low woven bone cells. **(B)** Box plot distribution of SpatialTime for pseudotime-high (orange) and pseudotime-low (red) woven bone spatial spots. **(C)** *Col10a1* expression among pseudotime-high (orange) and pseudotime-low (red) woven bone spatial spots.

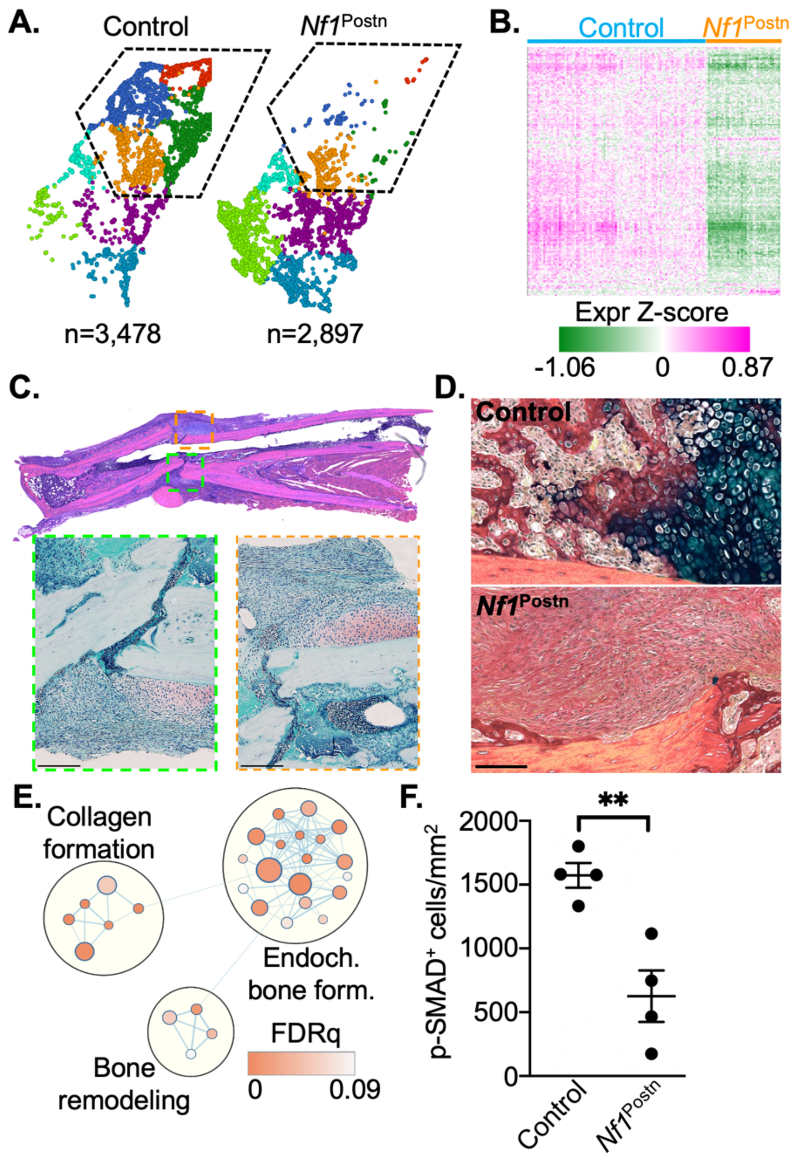

Supplemental Figure 3. **Spatial transcriptome analysis of a *Nf1*^{Postn} fracture callus. (A)** UMAP of spatial clusters from control and *Nf1*^{Postn} fracture calluses. Dashed line outlines skeletal clusters involved in the reparative process after fracture. **(B)** Heatmap of core genes (from Figure 4B) in skeletal clusters from *Nf1*^{Postn} fracture sections compared to control. **(C)** H/E (*top*) and Safranin O (*bottom*) staining of the *Nf1*^{Postn} fracture. Scale bar = 200µm. **(D)** Picrosirius red/alcian blue stain of control and *Nf1*^{Postn} fracture. Scale bar = 100µm. **(E)** Enrichment map graphically summarizing endochondral molecular pathways with

significantly reduced expression in the cortical bone clusters from *Nf1*^{Postn} mice compared to control. **(F)** Quantification of pSmad^{+} cells per periosteal area in the control and *Nf1*^{Postn} fracture. Data represent mean ± SEM from four replicate measurements per group.

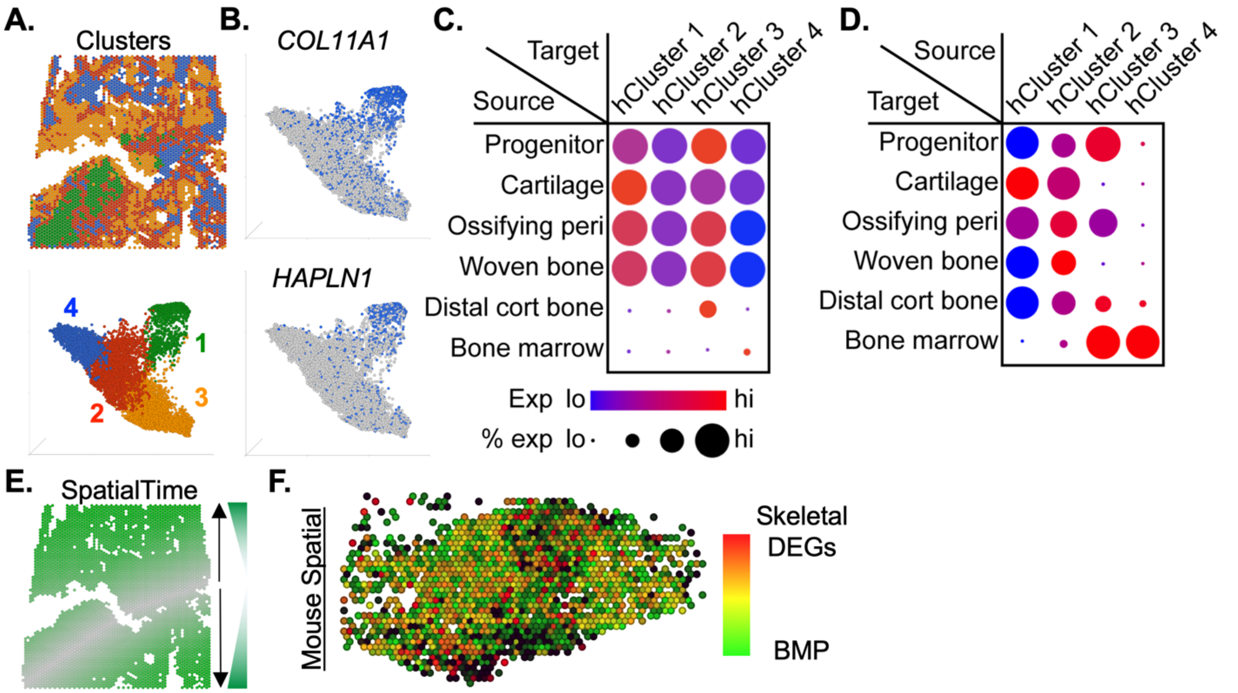

Supplemental Figure 4. **Analysis of human pseudarthrosis spatial clusters. (A)** Spatial distribution of spatial clusters (*top*) and UMAP plot (*bottom*) of spatial clusters from human fracture pseudarthrosis sections. **(B)** UMAP expression of *COL11A1* (*top*) and *HAPLN1* (*bottom*). **(C,D)** Bubble plot comparing the relative expression of (c) mouse cluster-associated genes in human spatial clusters or (D) human cluster-associated genes in mouse spatial clusters. **(E)** Spatial distribution of SpatialTime within the human fracture pseudarthrosis section. **(F)** Blended feature plot demonstrating co-localization of BMP pathway genes and orthologous down-regulated human skeletal DEGs (from Figure 5A) in the control mouse fracture callus.
